# Low Risk for Developing Diabetes Among the Offspring of Individuals With Exceptional Longevity and Their Spouses

**DOI:** 10.3389/fcdhc.2022.753986

**Published:** 2022-04-11

**Authors:** Iva Miljkovic, Ryan Cvejkus, Ping An, Bharat Thyagarajan, Kaare Christensen, Mary Wojczynski, Nicole Schupf, Joseph M. Zmuda

**Affiliations:** ^1^ Department of Epidemiology, Graduate School of Public Health, University of Pittsburgh, Pittsburgh, PA, United States; ^2^ Division of Statistical Genomics, Department of Genetics, Washington University School of Medicine, St. Louis, MO, United States; ^3^ Department of Laboratory Medicine and Pathology, School of Medicine, University of Minnesota, Minneapolis, MN, United States; ^4^ Department of Epidemiology, Biostatistics and Biodemography, Danish Aging Research Center, University of Southern Denmark, Odense, Denmark; ^5^ Taub Institute, Columbia University, New York, NY, United States

**Keywords:** diabetes, longevity, offspring, long-living individuals, Family Study

## Abstract

Little is known about the risk of type 2 diabetes (T2D) among the offspring of individuals with exceptional longevity. We determined the incidence of and potential risk and protective factors for T2D among the offspring of probands and offspring’s spouses (mean age=60 years, range 32-88 years) in the Long Life Family Study (LLFS), a multicenter cohort study of 583 two-generation families with a clustering of healthy aging and exceptional longevity. Incident T2D was defined as fasting serum glucose ≥126 mg/dl, or HbA1c of ≥6.5%, or self-reported with doctor diagnosis of T2D, or the use of anti-diabetic medication during a mean follow-up 7.9 ± 1.1 years. Among offspring (n=1105) and spouses (n=328) aged 45-64 years without T2D at baseline visit, the annual incident rate of T2D was 3.6 and 3.0 per 1000 person-years, respectively, while among offspring (n=444) and spouses (n=153) aged 65+ years without T2D at baseline, the annual incident rate of T2D was 7.2 and 7.4 per 1000 person-years, respectively. By comparison, the annual incident rate of T2D per 1000 person-years in the U.S. general population was 9.9 among those aged 45-64, and 8.8 among those aged 65+ years (2018 National Health Interview Survey). Baseline BMI, waist circumference, and fasting serum triglycerides were positively associated with incident T2D, whereas fasting serum HDL-C, adiponectin, and sex hormone binding globulin were protective against incident T2D among the offspring (all P<0.05). Similar associations were observed among their spouses (all P<0.05, except sex hormone binding globulin). In addition, we observed that among spouses, but not offspring, fasting serum interleukin 6 and insulin-like growth factor 1 were positively associated with incident T2D (P<0.05 for both). Our study suggests that both offspring of long-living individuals and their spouses, especially middle-aged, share a similar low risk for developing T2D as compared with the general population. Our findings also raise the possibility that distinct biological risk and protective factors may contribute to T2D risk among offspring of long-lived individuals when compared with their spouses. Future studies are needed to identify the mechanisms underlying low T2D risk among the offspring of individuals with exceptional longevity, and also among their spouses.

## Introduction

Preserved glucose tolerance and insulin sensitivity has been recognized as one of the major biological pathways to maintaining health and achieving exceptional longevity (1) (2). Comparisons of nonagenarians and centenarians with “younger” individuals aged 65-89 years from the same study indicate that improved life expectancy is consistently associated with favorable fasting serum glucose and preserved insulin sensitivity (3–8). Previous reports have also shown that centenarians may have preserved glucose tolerance even comparable with that of healthy young individuals (9). However, little is known about the incidence of and risk or protective factors for type 2 diabetes (T2D) among the offspring of individuals with exceptional longevity. Lifestyle, environmental, and genetic factors likely contribute to the complex phenotype of exceptional longevity. Although, several cross-sectional studies have reported a low prevalence of T2D among the offspring of individuals with exceptional longevity (10–12), similar prospective studies are lacking. When studying offspring of individuals with exceptional longevity, it is a challenge to select a proper comparison group that may adequately represent the general population that also have measured important confounders. The spouses of the offspring could offer an advantageous approach as they are expected to be close in age, and matched on environment, socioeconomic and geographical background (11, 13, 14). Interestingly, previous studies have reported that the mortality of spouses marrying into longevity-enriched families is substantially lower than the mortality in the general population (15).

The Long Life Family Study (LLFS) is a multicenter cohort study of two-generation families with a clustering of healthy aging and exceptional survival. We have previously shown that diabetes prevalence was lower and glucose metabolism seem to be healthier in LLFS probands and offspring as well as their spouses compared to similar aged persons in the other epidemiologic cohorts (16), making the LLFS a valuable longitudinal cohort to study the glucose metabolism and diabetes and how they relate to longevity. Our primary objective was to compare the rate of T2D incidence among the offspring of LLFS probands and offspring spouses, as well as compare these rates with the rates observed in general population. Our secondary objective was to identify potential risk and protective factors for developing T2D among the offspring and compare them with their spouses.

## Methods and Methods

### Study Population: The Long-Life Family Study

The Long-Life Family Study (LLFS) is a family-based cohort study of exceptional longevity that recruited families at four study centers (Boston, Massachusetts; New York, New York; Pittsburgh, Pennsylvania; and Denmark). The three U.S. field centers used Center for Medicare and Medicaid Services lists of Medicare enrollees to mail a recruitment brochure. The initial file included people who were at least 79 years old on January 1, 2005; had no recorded date of death; were not in the end-stage renal disease or hospice programs; and lived in zip codes within 3 hours driving distance one of the study centers. A pilot mailing tested the yield of families recruited from mailing to individuals in their 80’s and higher age strata. Based on these yields, subsequent mailings targeted those age 89 and older. Study participants were also recruited from local communities using mailed brochures, posters, web-based media and newspaper advertisements. Additional mailing lists were obtained through voter registries and purchased public domain lists from various commercial vendors. The University of Southern Denmark field center identified individuals who would be ages 90 and above during the study recruitment period through the Danish National Register of Persons, which contains current information on names, including past names such as maiden names for women, addresses, place of birth, marriages, and vital status (17). Second, using information on the place of birth and the names, parish registers available in regional archives were searched to locate the parents of the elderly individuals in order to identify sibships. Based on the above information, potentially eligible families were identified, and contact was made with potential probands to further assess the family’s eligibility for and willingness to participate in the LLFS using criteria parallel to that used in the United States. The criteria for the final recruitment were based on having 2 or more siblings who were exceptionally long-lived (aged 80+ years in the US and 90+ years in Denmark). Families were primarily white and met the following eligibility criteria: 1) enrolled one long-lived participant (proband) aged ≥90, 2) enrolled ≥1 sibling of the proband, 3) enrolled ≥1 offspring of either the proband or the proband’s sibling, and 4) the proband generation had a clustering of members with exceptional survival based on a family longevity selection score (18). Briefly, the Family Longevity Selection Score (FLoSS) was used to rank a potential proband sibship on their combined exceptionality of survival (18). A family’s entry into LLFS required at least one living member of the proband sibship with “decisional capacity”, a living offspring, and a proband sibship FLoSS of at least 7. The FLoSS is designed to be negative for families with less than average longevity, with higher scores representing increasingly exceptional longevity. For example, the FLoSS for a five-person sibship with each sib at the 91st percentile of longevity for his or her birth cohort is about 7; and if all five sibs were at the 98th percentile, its FLoSS would be nearly 15. As an indication of the exceptionality of LLFS sibships, fewer than 1% of families in the Framingham Heart Study sample have a FLoSS > 7 (18). The two generations in the LLFS were labeled as the proband generation (long-lived individual and their enrolled siblings) and the offspring generation (all enrolled offspring of individuals in the proband generation). The LLFS also recruited as many spouses as possible. Spouses of the proband generation were recruited only if their biological children were enrolled in the study. Spouses of the offspring generation were recruited as spousal controls, if they lived in the same household as their offspring pair. In total, 4559 long-lived probands and their siblings (n=1445), their offspring (n=2329) and spouse controls (n=785) were recruited from 2006 to 2009. Other characteristics of family eligibility, recruitment, and composition have been previously described (16, 18).

From 2014 to 2017, surviving participants were invited to take part in an in-home follow-up examination. In total, 3,198 individuals participated in the follow-up exam (86% of survivors). Within the offspring generation (including spousal controls), 3,114 individuals participated in the baseline exam, and 2,219 individuals participated in the follow-up exam (74.4% of survivors).

Written informed consent was obtained from each LLFS participant using forms and procedures approved by each participating institution’s Institutional Review Board.

### Interview and Measurements

Sociodemographic factors, including date of birth, gender, race, and education, smoking status, difficulty with activities of daily living, health status, and chronic conditions were determined by interview, in addition a blood sample was collected, in the participant’s home at both visits. History or presence of type 2 diabetes, heart disease, stroke, cancer, emphysema, and chronic obstructive pulmonary disease was based on self-report of a physician’s diagnosis. All prescription and non-prescription medications were examined in their original containers for a medication inventory. Weight, waist circumference, and systolic and diastolic blood pressure were measured at both visits.

Biological markers evaluated in the current analysis included: lipids (triglycerides, high-density lipoprotein cholesterol (HDL-C), low-density lipoprotein cholesterol (LDL-C), total cholesterol), pro- and anti- inflammatory biomarkers (adiponectin, interleukin 6, high-sensitivity C-reactive protein), insulin-like growth factor 1 (IGF-1), and sex hormone binding globulin (SHBG). All biomarkers were measured in fasting blood by a central laboratory at the University of Minnesota. Participants were asked to fast for at least eight hours prior to the blood draw.

T2D was defined as fasting serum glucose ≥126 mg/dl, or glycated hemoglobin (HbA1c) of ≥6.5%, or self-reported with doctor diagnosis of T2D, or the use of anti-diabetic medication, and incident T2D was determined during a mean follow-up 7.9 ± 1.1 years.

### Statistical Analysis

Baseline and metabolic characteristics of offspring and their spousal controls were summarized and compared using age- and sex- adjusted means and standard errors for continuous traits, and frequencies and percent for categorical traits. Each trait was assessed for normality and transformed as needed before statistical comparison.

The associations of each of these traits with odds of incident T2D were determined in the offspring group using generalized linear models, incorporating a logit link and an exchangeable correlation structure within families to account for genetic relatedness of individuals. Associations of incident T2D were determined in the spousal control group using multiple logistic regression. Covariates included in the models were age, sex, BMI, field center, and lifestyle factors including alcohol intake and physical activity. These covariates were determined *a priori* and were forced into each model. Odds ratios of incident diabetes are presented per standard deviation of each continuous trait or per level of each categorical trait, in offspring and their spouses separately.

## Results

### Baseline Characteristics

For the current analyses, we focused on baseline offspring and their spouses without T2D only. 2,889 non-diabetics participated in the baseline exam and 2,080 of those individuals also participated in the follow-up exam (74.9% of survivors). Offspring and their spouses who participated in the follow-up exam were more likely to be women, physically active, and to consume alcohol, and less likely to be obese compared with those who did not participate in the follow-up (all P<0.05, data not shown).

**Table 1** shows selected baseline characteristics of LLFS offspring and their spouses without T2D who returned for Exam 2. Offspring and their spouses were on average 60 years old. At baseline, offspring were less likely to be men (42% *vs* 52%, P<0.0001), to be married (75.4% *vs* 98.9%, P<0.001), to drink four of more alcoholic drinks per week (42.1% *vs* 54.1%, P<0.0001), and to walk three or more hours per week (76.7% *vs*. 81.1%, P=0.016, but more likely to achieve a high school or higher education than their spouses (91.1% *vs*. 94.5%, P=0.0036) Offspring also had slightly lower level of fasting plasma LDL-C at baseline compared to their spouses (123.6 *vs*. 127 mg/dl, P=0.046).

**Table 1:** Selected baseline characteristics of LLFS offspring and their spouses without T2D.

Mean ± SD	Offspring (n=1585)	Offspring Spouses (n=495)	p-value*
Median [Q1, Q3]			
Men	679 (42.8%)	260 (52.5%)	**<0.0001**
Age (years)	60.1 ± 8.0	60.5 ± 8.3	0.46
(range)	(32–88)	(36–88)	
BMI (kg/m2)	27.0 ± 4.7	26.9 ± 4.3	0.33
Waist Circumference (cm)	93.0 ± 13.3	93.8 ± 12.2	0.36
Married	1195 (75.4%)	488 (98.9%)	**<0.0001**
High School Education or Higher	1497 (94.5%)	450 (91.1%)	**0.0036**
Current Smoker	128 (8.1%)	43 (8.7%)	0.89
Drinks per Week	666 (42.1%)	266 (54.1%)	
≥ 4			**<0.0001**
Walking hours per week	1208 (76.7%)	400 (81.1%)	
≥ 3			**0.016**
Triglycerides (mg/dl)**	90.0 [66.0, 130.0]	94.0 [67.0, 133.0]	0.52
HDL (mg/dl)**	61.6 ± 17.7	59.5 ± 16.4	0.29
LDL (mg/dl)**	123.6 ± 33.0	127.0 ± 34.2	**0.046**
Adiponectin (ng/mL)	9977.0 [6580.0, 14408.0]	9208.0 [6296.0, 13005.0]	0.23
IL-6 (pg/mL)	0.7 [0.4, 1.1]	0.7 [0.5, 1.2]	0.71
hsCRP (mg/L)	1.2 [0.6, 2.4]	1.1 [0.5, 2.4]	0.46
IGF1 (ng/mL)	133.0 [105.0, 177.0]	130.0 [107.0, 170.0]	0.13
SHBG (nmol/L	53.0 [38.0, 75.0]	54.0 [39.0, 74.0]	0.22

Normally distributed continuous variables presented as unadjusted Mean ± SD, non-normal continuous variables presented as Median [IQR].

*P-values adjusted for Age and Sex.

**Adjusted in addition for relevant medication.

The bold value indicates a p < 0.05.

### Total Cumulative and Annualized T2D Incidence

Among offspring (n=1585) and spouses (n=495) without diabetes at study entry, 58 (3.7%) and 19 (3.8%) developed incident T2D, respectively. Annual incidence rate was 4.6 cases per 1000 person-years among the offspring, and 4.7 cases per 1000 person-years among the spousal controls. Among offspring (n=1105) and spouses (n=328) without diabetes and aged 45-64 years the annual incident rate of T2D per 1000 person-years was 3.6 and 3.0, respectively, while among offspring (n=444) and spouses (n=153) without diabetes and aged 65 years and older at study entry, the annual incident rate of T2D per 1000 person-years was 7.2 and 7.4, respectively. There was no difference between the T2D incidence rate in the U.S. Study Centers *vs*. Denmark, in offspring nor spouses in the total sample or by age groups (data not shown).

### Risk Factors for Incident T2D in Offspring and Their Spouses

**Table 2** present the odds of incident T2D per standard deviation greater level of baseline risk factor for continuous variables or presence of the risk factor for dichotomous variables. BMI (OR=2.55, P<0.0001), waist circumference (OR=2.52, P<0.0001), and fasting serum triglycerides (OR=1.56, P=0.0042), at baseline visit were positively, whereas HDL-C (OR=0.56, P<0.0042), adiponectin (OR=0.60, P<0.0024), and sex hormone-binding globulin (OR=0.55, P<0.0019), were inversely associated with incident T2D among the offspring. Similar associations were observed in their spouses, although there was no significant association between sex hormone-binding globulin and incident T2D (**Table 3**). Additionally, among the offspring spouses, we observed that circulating interleukin 6 (OR=1.63, P=0.048) and insulin-like growth factor 1 (OR=1.79, P=0.04) were positively associated with incident T2D (**Table 3**). Among both the offspring and their spouses none of the lifestyle factors measured in our study were associated with incident T2D.

**Table 2:** Risk factors for incident T2D in LLFS offspring^+^.

Baseline Risk Factors	SD	Odds of Developing	Multi-variable adjusted
		T2D	P Value
Age (years)	12.2	1.25	0.25
BMI (kg/m2)*	4.7	**2.55**	**<0.0001**
Waist Circumference (cm)*	13.3	**2.52**	**<0.0001**
Triglycerides (mg/dl)**	72.9	**1.56**	**0.0042**
HDL (mg/dl)**	17.3	**0.56**	**0.03**
LDL (mg/dl)**	34.4	0.85	0.45
SBP (mmHg)**	20.5	0.90	0.49
DBP (mmHg)**	10.7	0.90	0.50
Adiponectin (ng/mL)	6780	**0.60**	**0.0024**
IL-6 (pg/mL)	4.4	1.14	0.44
hsCRP (mg/L)	6.1	1.20	0.11
IGF1 (ng/mL)	54.4	0.96	0.79
SHBG (nmol/L)	35.2	**0.55**	**0.0019**
Alcoholic intake (4+ drinks per week)	Yes/No	0.71	0.32
Walking (3+ hours per week)	Yes/No	0.64	0.15
Current Smoker	Yes/No	1.10	0.83

+Risk is the odds of Incident T2D per 1 SD greater level of baseline risk factor for continuous variables or presence of the risk factor for dichotomous variables.

Adjusted for age, sex, BMI, lifestyle factors, field center, and family structure.

*Not adjusted for BMI.

**Additionally adjusted for relevant medication.

The bold value indicates a p < 0.05.

**Table 3:** Risk factors for incident T2D in LLFS spousal controls^+^.

Baseline Risk Factors	SD	Odds of Developing	Multi-variable adjusted
		T2D	P Value
Age (years)	12.2	1.26	0.53
BMI (kg/m2)*	4.7	**1.9**	**0.0073**
Waist Circumference (cm)*	13.3	**2.1**	**0.007**
Triglycerides (mg/dl)**	72.9	1.63	0.055
HDL (mg/dl)**	17.3	**0.43**	**0.026**
LDL (mg/dl)**	34.4	1.27	0.37
SBP (mmHg)**	20.5	1.21	0.41
DBP (mmHg)**	10.7	1.14	0.56
Adiponectin (ng/mL)	6780	**0.54**	**0.012**
IL-6 (pg/mL)	4.4	**1.63**	**0.048**
hsCRP (mg/L)	6.1	0.98	0.95
IGF1 (ng/mL)	54.4	**1.79**	**0.04**
SHBG (nmol/L)	35.2	0.59	0.10
Alcoholic intake (4+ drinks per week)	Yes/No	0.99	0.98
Walking (3+ hours per week)	Yes/No	0.95	0.93
Current Smoker	Yes/No	1.68	0.51

+Risk is the odds of Incident T2D per 1 SD greater level of baseline risk factor for continuous variables or presence of the risk factor for dichotomous variables.

Adjusted for age, sex, BMI, lifestyle factors, and field center.

*Not adjusted for BMI.

**Additionally adjusted for relevant medication.

The bold value indicates a p < 0.05.

## Discussion

We (16) and others (8, 19) have previously shown that individuals with exceptional longevity and their offspring exhibit a healthier metabolic profile including glycemic control when compared with the general population. A surprising finding from the Long Life Family Study is a marked survival advantage among spouses to offspring of long-lived families when compared with the general population (15, 20). We have hypothesized that the risk of developing incident T2D among the offspring of long-lived individuals would be similar to the risk among their spouses, and lower than the risk in the general population. Indeed, we found that both offspring and their spouses, especially middle-aged, may share a similar, low rate of T2D, which is lower than the rates observed in general adult population from the most recent National Health Interview Survey (21). Specifically, the U.S. National Health Interview Survey (21) reports T2D incidence as 4.3 per 1000 person-years among individuals aged 18-44 years, 9.9 per 1000 person-years among those aged 45-64 year, 8.8 per 1000 person-years in those 65 and older years, and 6.9 per 1000 person-years among their total sample (individuals aged 18 years and older). While hereditary influences, such as genetic and/or epigenetic mechanisms, are likely to play a significant role in T2D risk (22), shared environmental factors are also likely to contribute to our findings. Compatibility in lifestyle (23–26) and leisure preferences (27) among couples who live together have been previously documented. In our study, spouses were actually more likely to be physically active and to report moderate alcohol consumption when compared with the offspring, and both of these lifestyle factors may reduce T2D risk (28–30). Thus, it is possible that the protective familial genetic and biological factors impact glucose homeostasis among the offspring, whereas in their spouses the healthier lifestyles may in fact contribute to their lower T2D risk. Another possible explanation might be assortative mating - a nonrandom mating and sexual selection based on similar phenotypes and henceforth similar genotypes. It has been previously proposed that assortative mating of members from families enriched for longevity could preserve longevity across generations by increasing the likelihood of transmission of rare variants with a recessive effect (31). Further studies in the offspring and their spouses in the LLFS could help us better understand a lower risk of T2D associated not only with being an offspring of a long-lived individuals, but also with being married into a long-lived family.

A second objective of our analysis was to identify potential risk and protective factors for T2D among the offspring of long-living individuals, and to compare these factors to those in spouses. Significant associations between the anthropometric measures, lipid and lipoproteins, and adiponectin and incident T2D were very similar among the offspring and their spouses. However, our findings raise the possibility that biological factors contributing to T2D might be different in the offspring as compared to their spouses. We found that pro-inflammatory and insulin-like growth factor signaling biomarkers may play a greater role in T2D among spouses than in the offspring of the exceptional survivors. In contrast, sex hormone-binding globulin might be uniquely protective against T2D among the offspring of exceptional survivors. Further research is needed to identify the molecular mechanisms and pathways that are underlying this low T2D risk and favorable glucose control in offspring of exceptionally long-lived individuals and their spouses, as the LLFS is currently generating transcriptomic, methylomic, proteomic, and metabolomic data longitudinally.

The biology underlying the association between glucose metabolism and longevity is still under extensive investigation, but lipid and lipoprotein metabolism (32) and adipokine signaling pathways (33, 34) have emerged as a possible mechanistic link. In our study, greater fasting serum TG and lower fasting serum HDL-C and adiponectin appear to have similar relationships with T2D in offspring and their spouses. Dysregulation of adipokines is associated with insulin resistance, hyperglycemia, dyslipidemia, and T2D (35), as well as with wasting syndromes, such as cachexia (36), suggesting that adipose tissue endocrine function might be essential for maintaining whole-body energy homeostasis with aging. Furthermore, genetic manipulation of adipose tissue promotes longevity in mice, suggesting a possible role in longevity (37). We did not have measures of body composition in the current study to address the role of adipose tissue. Our findings suggest that the offspring of long-living individuals might be protected against the detrimental effects of IL-6 and IGF-1 on the risk of T2D, in contrast to their spouses. The observed association between IL-6 and incident T2D in spousal controls is consistent with a recent meta-analysis of 15 prospective studies which reported that higher levels of IL-6 were significantly associated with a higher risk of incident T2D (38). The link between IL-6 and longevity is less explored, though some studies suggest that greater circulating IL-6 is associated with a higher risk of mortality, including among successfully aging individuals (39). Although, it is known that IGF-1 is involved in glucose metabolism (40), prospective studies have been inconclusive, some reporting no association between serum IGF-1 and the risk of T2D (41), while others found positive (42, 43) or even inverse (44) associations. In animal models, down-regulation of the GH/IGF-1/insulin system significantly prolongs lifespan, but data in humans are inconsistent (45). Finally, low circulating level of the sex hormone-binding globulin is another well-known predictor of the risk of T2D (46). In our study, sex hormone-binding globulin was protective against T2D, but only among the offspring of long-lived individuals. The link between sex hormone-binding globulin and longevity is still not well understood (47–49).

There are several limitations of our study. Our analyses are potentially limited by the smaller sample size of the spousal control group, and the fact that family history of longevity was not collected for spousal controls. It is possible that some of the spousal controls may have come from long-lived families. Our cohort included only European ancestry individuals, and thus, findings may not apply to other ethnic/race groups. Furthermore, data on dietary intake and dietary patterns were not collected and specific foods and overall dietary patterns may be associated with T2D risk. Additionally, biomarkers were only measured at baseline. Finally, we only studied several candidate biomarkers, which were available in our study, but there are many other relevant biological factors which may be associated with T2D. On the other hand, a major strength of our study is that it includes the spouses of the offspring. By sharing the same environment, it is less likely that environmental factors would have confounded the observed differences between the offspring and their spouses. However, although our records indicate that the spouses shared the physical address with their offspring pair, it is possible that they were spending some time apart, and thus, might have not shared the same environment all the time.

In conclusion, our study suggests that offspring of exceptionally long-lived individuals and their spouses, especially at middle-age, may share a similar, low risk for developing T2D compared with the general population. Our findings also raise the possibility that distinct biological risk factors may contribute to T2D risk among offspring of exceptional survivors compared with their spouses. Additional studies are needed to identify the biological mechanisms underlying low T2D risk among the offspring of exceptionally long-lived individuals, but also among their spouses.

## Data Availability Statement

The raw data supporting the conclusions of this article will be made available by the authors, without undue reservation.

## Ethics Statement

The studies involving human participants were reviewed and approved by each participating institution’s Institutional Review Board. The patients/participants provided their written informed consent to participate in this study.

## Author Contributions

Manuscript concept: IM and JZ. Data analysis: RC. Manuscript writing: IM. Interpretation of data and manuscript editing and critical review: all authors. All authors contributed to the article and approved the submitted version.

## Funding

National Institute on Aging-National Institutes of Health (Grants U01-AG023746, U01-AG023712, U01-AG023749, U01-AG023755, U01-AG023744, and U19 AG063893).

## Conflict of Interest

The authors declare that the research was conducted in the absence of any commercial or financial relationships that could be construed as a potential conflict of interest.

## Publisher’s Note

All claims expressed in this article are solely those of the authors and do not necessarily represent those of their affiliated organizations, or those of the publisher, the editors and the reviewers. Any product that may be evaluated in this article, or claim that may be made by its manufacturer, is not guaranteed or endorsed by the publisher.
